# Computational modelling of wounded tissue subject to negative pressure wound therapy following trans-femoral amputation

**DOI:** 10.1007/s10237-017-0921-7

**Published:** 2017-05-28

**Authors:** B. Zeybek, S. Li, J. W. Fernandez, S. Stapley, V. V. Silberschmidt, Y. Liu

**Affiliations:** 1 Wolfson School of Mechanical Electrical and Manufacturing Engineering, Loughborough University, Loughborough, UK; 2 Auckland Bioengineering Institute, Auckland University, Auckland, New Zealand; 3 Department of Engineering Science, Auckland University, Auckland, New Zealand; 4 Royal Centre for Defence Medicine, ICT Centre, Birmingham, UK; 5 Department of Trauma and Orthopedics, Queen Alexandra Hospital, Portsmuth, UK; 6 The Centre of Biological Engineering, Loughborough University, Loughborough, UK

**Keywords:** Finite-element analysis, Biomechanics, Negative pressure wound therapy, Tissue oxygenation

## Abstract

Proof-of-concept computational models were developed and applied as tools to gain insights into biomechanical interactions and variations of oxygen gradients of wounded tissue subject to negative pressure wound therapy (NPWT), following trans-femoral amputation. A macro-scale finite-element model of a lower limb was first developed based on computed tomography data, and distributions of maximum and minimum principal stress values we calculated for a region of interest (ROI). Then, the obtained results were applied iteratively as new sets of boundary conditions for a specific spatial position in a capillary sub-model. Data from coupled capillary stress and mass- diffusion sub-models were transferred to the macro-scale model to map the spatial changes of tissue oxygen gradients in the ROI. The −70 mmHg NPWT resulted in a dramatic change of a wound surface area and the greatest relative contraction was observed at −150 mmHg. Tissue lateral to the depth of the wound cavity revealed homogenous patterns of decrease in oxygenation area and the extent of such decrease was dependent on the distance from the wound surface. However, tissue lateral to the width of the wound demonstrated heterogeneous patterns of change, as evidenced by both gradual increase and decrease in the oxygenation area. The multiscale models developed in the current study showed a significant influence of NPWT on both macro-deformations and changes of tissue oxygenation. The patterns of changes depended on the depth of the tissue, the geometry of the wound, and also the location of tissue plane.

**Electronic supplementary material:**

The online version of this article (doi:10.1007/s10237-017-0921-7) contains supplementary material, which is available to authorized users.

## Introduction

The care of complex wounds is one of the most significant challenges for healthcare systems today. In an ideal scenario, the residual extremity is covered with well-vascularized muscle, fascia, and skin. However, in the case of traumatic amputation, the remaining skin structure is not always sufficient to fully cover the wounded area and it is therefore difficult to dress (Hinck et al. 2010; Couch and Stojadinovic 2011; Richter and Knudson 2013a; Armstrong et al. 2016). The use of negative pressure wound therapy (NPWT) in war wounds was first reported in 2004, and it represents a new approach in war wound reconstruction (Bhandari et al. 2012). In recent years, NPWT has become an accepted option for managing and treating trauma cases (Kanakaris et al. 2007). It is also beginning to be appreciated more widely in management of both high- and low-energy trauma wounds and open fractures of the lower extremity because of its ability to handle high volumes of exudate and provide a closed wound environment (Jeffery 2009).

The concept that the physiological, cellular, and molecular mechanisms induced by NPWT accelerate wound healing is now gradually starting to be understood (Banwell and Musgrave 2004; Orgill et al. 2009; Peinemann and Sauerland 2011; Huang et al. 2014). Preclinical and clinical studies have shown that NPWT provides a moist wound healing environment, drains exudate (Huang et al. 2014), reduces tissue oedema, contracts wound edges (Malmsjö et al. 2009a; Birke-Sorensen et al. 2011), mechanically stimulates the wound bed (Banwell and Musgrave 2004; Borgquist and Gustafsson 2010), alters blood flow in and around the wound edges (Malmsjö et al. 2009a), and stimulates angiogenesis and the formation of granulation tissue (Erba et al. 2011; Huang et al. 2014).

The mechanism for the increase in vascularisation is not totally understood but may be attributed to hypoxia-mediated angiogenesis (Orgill and Bayer 2011; Al-Shammari et al. 2014). Kairinos et al. also found that blood flow was dependent on the pressure applied, the distance from the edge, and the tissue type (Kairinos et al. 2009). Most of these experimental studies were conducted with a laser Doppler device to measure the blood flow rate. However, Kairinos et al. further pointed out the design flaw of such an experimental approach on understanding the influence of NPWT on blood perfusion, in particular, when the change of the geometry of lumen that delivers the blood had not been considered (Kairinos et al. 2014).

Collective findings from published work indicated that intrinsic and extrinsic factors related to wound pressure applied in those preclinical studies could influence the experimental outcomes as evidenced by the discrepancy observed between some studies (Morykwas et al. 1997; Wackenfors et al. 2004; Ichioka et al. 2008; Malmsjo et al. 2009). Simplified computational algorithms based on preclinical study data, using finite-element analysis, had been applied to predict the strains and microdeformations at the tissue and filler interface during the NPWT (Saxena and Hwang 2004; Scherer 2008; Wilkes et al. 2009, 2012). It appears that little work has been done about basic computational research on mechanical loads delivered to the wound area during the NPWT and there are no three-dimensional models of lower limbs, investigating the effect of NPWT treatment on tissue oxygenation. Accordingly, the main purpose of this study is to characterise mechanical deformations developed within the lower limb, with a particular focus on the mechanical loads delivered to the tissue and the corresponding pressure stress effects on tissue oxygenation around the wound area. To the best of our knowledge, this work presents the first multiscale computational approach to the investigation of tissue oxygenation changes in response to mechanical deformations caused by the topical application of negative pressure.

## Materials and methods

### Framework outline of multiscale computational approach

**Fig. 1:**
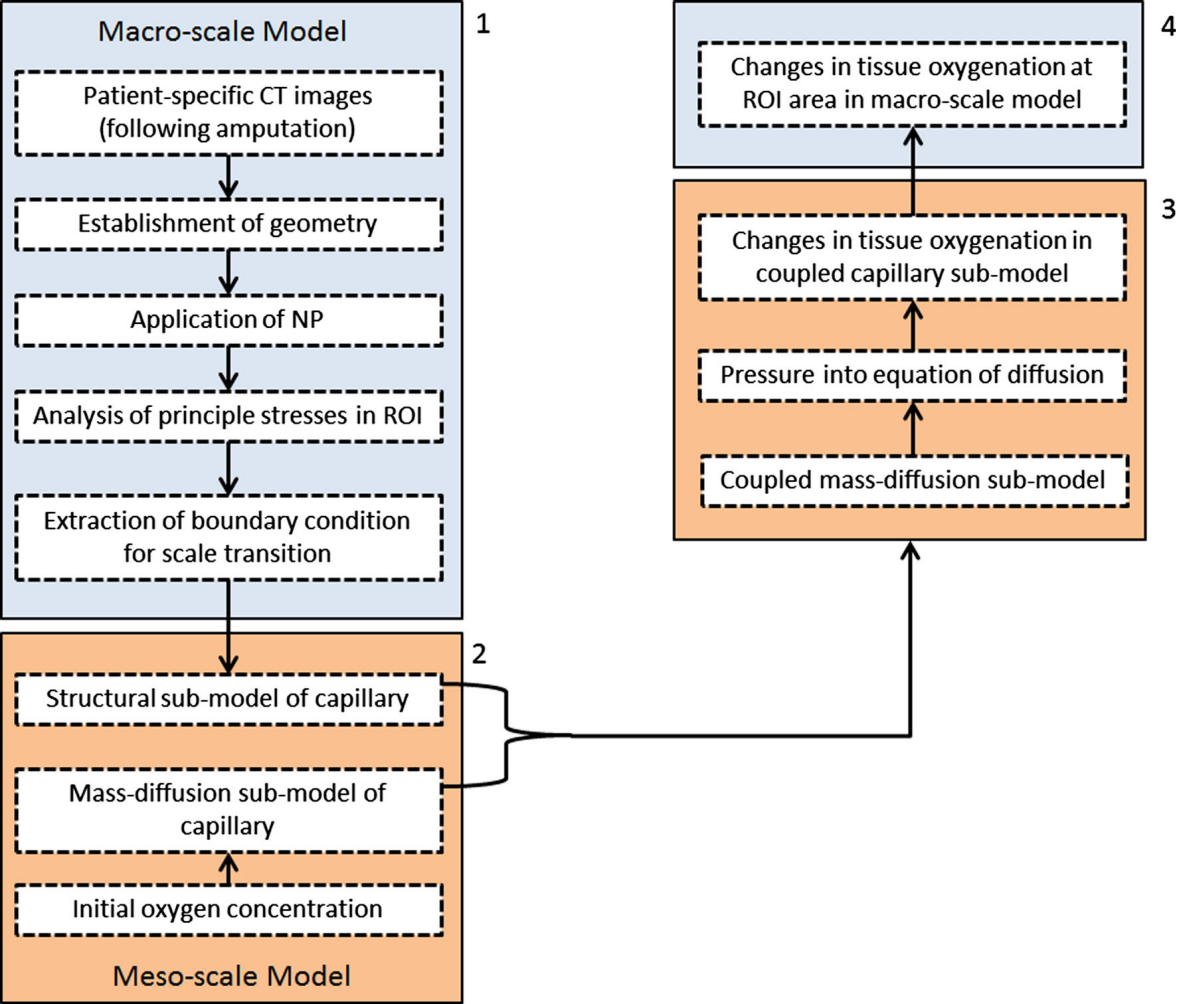
Schematic of computational framework: (*1*) macro-scale model of lower limb; (*2*) meso-scale sub-model of capillary; (*3*) coupling meso-scale structural model with meso-scale mass-diffusion model; (*4*) data feedback for macro-scale model for assessment of tissue oxygenation changes around wound bed

A three-dimensional finite-element (FE) model of the residual limb was developed, incorporating both the muscle and the bone, to analyse the application of negative pressure therapy. An overview of the framework illustrated in Fig. 1 shows the flow of information including (1) a macro-scale model of a lower limb, (2) a meso-scale sub-model of a capillary, (3) coupling the meso-scale structural model with a meso-scale mass-diffusion model, and (4) data feedback for assessment of macro-scale tissue oxygenation changes around the wound bed.

**Fig. 2:**
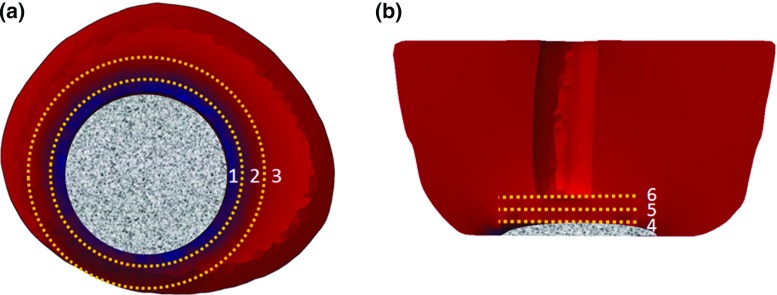
Schematic diagram of cross sections of model and definition of ROI areas around wound bed: **a** transversal cross section of macro-model; **b** frontal cross section of macro-model. The *dashed lines* labelled by numbers are correspond to tissue layers with different distances from the surface of the wound bed in the transversal and frontal directions: (*1*, *4*) 0 mm, (*2*, *5*) 10 mm, and (*3*, *6*) 15 mm

The macro-scale FE model, representing the entire structure of the residual limb, was used to obtain distribution of macroscopic stresses near the wound bed under the applied loading conditions of NPWT. Each element of the macro-scale model represented a volumetric region that defines direction-independent capillary sub-model in a physical domain. Distributions of maximum and minimum values of principal stress were calculated for the regions of interest (ROI) in the macro-scale model. ROI were parts of the muscle tissue at various depths from the wound bed surface. It was specified as layers of cross-sectional tissues with distance of 0, 10, and 15 mm from the surface of the wound bed in the transversal and frontal directions (see Fig. 2). Then, the outcomes of these calculations were applied to the capillary sub-models as sets of their boundary conditions for specific spatial positions within meso-scale analysis. Two capillary sub-models for stress and mass-diffusion analysis were coupled by scripting in the ABAQUS finite-element software package. Finally, these data were transferred to the macro-scale FE model to map the spatial changes of tissue oxygen distribution in the ROI. Details of the main elements in this framework are given in the subsequent sections.

**Fig. 3:**
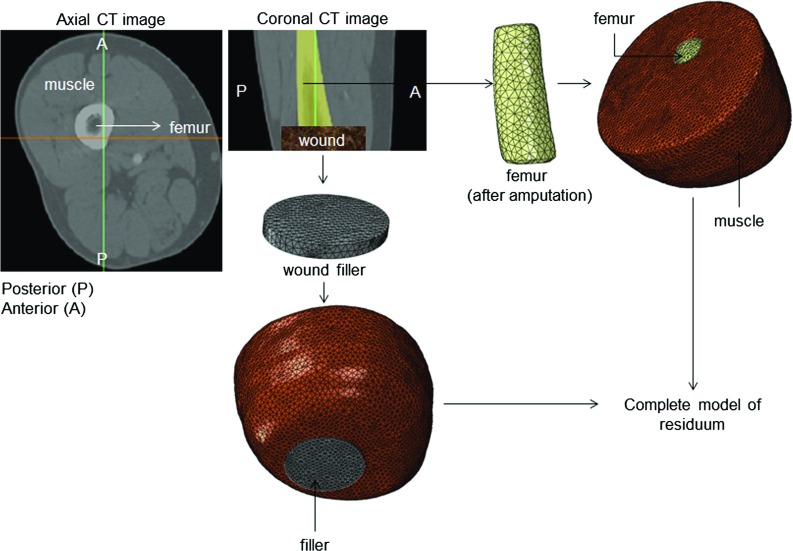
Complete patient-specific finite-element model of lower limb created from sets of axial CT scans

**Table 1:** List of material properties and respective equations used in macro- and meso-scale models, where $I_{1}$ and $I_{2}$ are the first and the second deviatoric strain invariants; $C_{1}$ and $C_{2}$ are the material constants characterizing deviatoric deformation of the material for Mooney–Rivlin strain-energy-density function

Model	Material	Material type and parameter	Parameters	References
Macro-scale model	Muscle tissue	Hyperelastic (Mooney–Rivlin)	$C_{1}(\hbox {MPa})=0.0094, C_{2}(\hbox {MPa})=0.082 D\,(\hbox {kg}/\hbox {mm}^{3})=0.00$	Portnoy et al. (2009)
	PU foam	Hyperfoam	$\mu =0.907\hbox {e}-2, \alpha =0.213\hbox {e}-2 \beta =0.844\hbox {e}-2$	Schrodt et al. (2005)
	Capillary wall	Hyperelastic (Mooney–Rivlin)	$C_{1}(\hbox {MPa})=0.257,\,\, C_{2}(\hbox {MPa})=0.00257 D\,(\hbox {kg}/\hbox {mm}^{3})=1\hbox {e-}06$	Huang et al. (2012)
Meso-scale model	Capillary surrounding muscle tissue	Hyperelastic (Mooney*–Rivlin)	$C_{1 }(\hbox {MPa})=0.0094,\,\,C_{2}(\hbox {MPa})=0.082 D\,(\hbox {kg}/\hbox {mm}^{3})=0.00$	Portnoy et al. (2009)
	Capillary surrounding muscle tissue	Diffusivity of oxygen	$D=2\times 10^{-5}\,\hbox {cm}^{2}/s$	Zhao and Iramina (2015)
	Capillary wall	Diffusivity of oxygen	$D=1\times 10^{-5}\,\hbox {cm}^{2}/s$	Zhao and Iramina (2015)
	Capillary surrounding muscle tissue	Solubility of oxygen	$s=3.89\times 10^{-5}\,\hbox {ml}\,\hbox {O}_{2}\,\hbox {ml}^{-1}\,\hbox {mmHg}$	Goldman et al. (2004)

	Equation		Description	Number
Respective equations	$W=C_{1}\left( {I_{1}-3}\right) +C_{2}\left( {I_{2}-3}\right)$		Mooney–Rivlin strain-energy-density function	(1)
	$p=-tr\,\sigma /3$		Equivalent pressure stress	(2)
	$J=-D\frac{\partial C}{\partial x}+sK_\mathrm{P} \frac{\partial p}{\partial x}$		Extended form of Fick’s law	(3)

In the extended form of the Fick’s law, *D* is the diffusivity; *C* is the mass concentration of the diffusing material; *s* is the solubility; $K_{P}$ is the pressure stress factor; and *p* is equivalent pressure stress. All governing equations were adopted from Abaqus 6.14 Documentation

### Macro-scale finite-element analysis model

A set of computed tomography (CT) scans, obtained from OsiriX DICOM Image Library, was used to obtain a more realistic geometry of a trans-femoral lower extremity. The three-dimensional (3D) geometry of the lower limb was reconstructed using image-segmentation software Mimics 19.0 (Materialise, Leuven, Belgium) following a standard segmentation procedure. The reconstructed computer-aided design (CAD) model consisted of three main components: a rigid bone structure, a muscle tissue, and a wound. A virtual amputation was performed to generate a representation of the trans-femoral residual limb, following a general amputation procedure. Virtual resection was performed at 18 cm distal to the greater trochanter and 12 cm above the knee (Erikson and James 1973). A virtual circumferential wound cavity with radius of 3.6 cm and depth of 0.8 cm (with approximately $40\,\hbox {cm}^{2}$ surface area) was generated at the distal region of the residual limb (Pattison et al. 2005; Jeffery 2009; Richter and Knudson 2013b). An identically sized wound filler modelled as polyurethane filler was placed at the wound cavity (Fig. 3).

The established 3D geometries of the residual limb were meshed with tetrahedral elements using 3-Matic (Materialize, Leuven, Belgium) with finer discretisation at the wound-filler interface and around bone edges and imported in ABAQUS 6.14 software (Dassault Systems Simulia Corp, Providence, Rl, USA). This produced a total of 172,693 linear tetrahedron hybrid four-node (C3D4H type) elements in the model.

For the present study, mechanical behaviour of muscle tissue was assumed to be nonlinear, hyperelastic, homogeneous, and isotropic. Using a Mooney–Rivlin (M–R) model based on a strain-energy-density function (see Eq. 1 from Table 1) was assigned for muscle (Portnoy et al. 2009). Bone was considered as rigid body during the simulation since it is orders of magnitude stiffer than the surrounding muscle tissue. The wound filler was modelled as polyurethane foam using a second-order Ogden’s hyperfoam material model (Schrodt et al. 2005; Orgill et al. 2009). All properties of the materials used in this study were obtained from literature and are listed in Table 1.

**Fig. 4:**
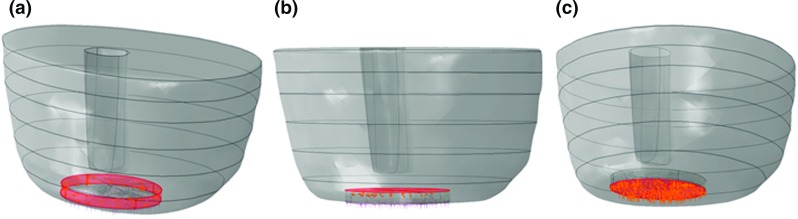
Schematic representation of simultaneously applied loading conditions for macro-scale model: The *red areas* represent the affected surface of tissue or filler, and *orange arrows* indicate the direction of applied pressure load. **a** Suction pressure to lateral face of the wound. **b** Suction pressure to the *bottom* of the wound and **c** pressure load to outer *top* of the filler

Different levels of negative pressure $70\,\hbox {mmHg} (0.0093\,\hbox {MPa}), 125\,\hbox {mmHg} (0.016\,\hbox {MPa})$, and $150\,\hbox {mmHg} (0.019\,\hbox {MPa})$ (Vig et al. 2011) were applied to represent suction within the wounded tissue area caused by the NPWT while a standard atmospheric pressure was applied to the top of the filler (Fig. 4). Tie-constrains was applied at bone–muscle tissue interface to represent the structural linkage between different layers; this ensured no slippage and a firm bond as a boundary condition. An encastre boundary condition was applied to the proximal end of the limb to constrain movement in all directions.

### Meso-scale capillary sub-models

**Fig. 5:**
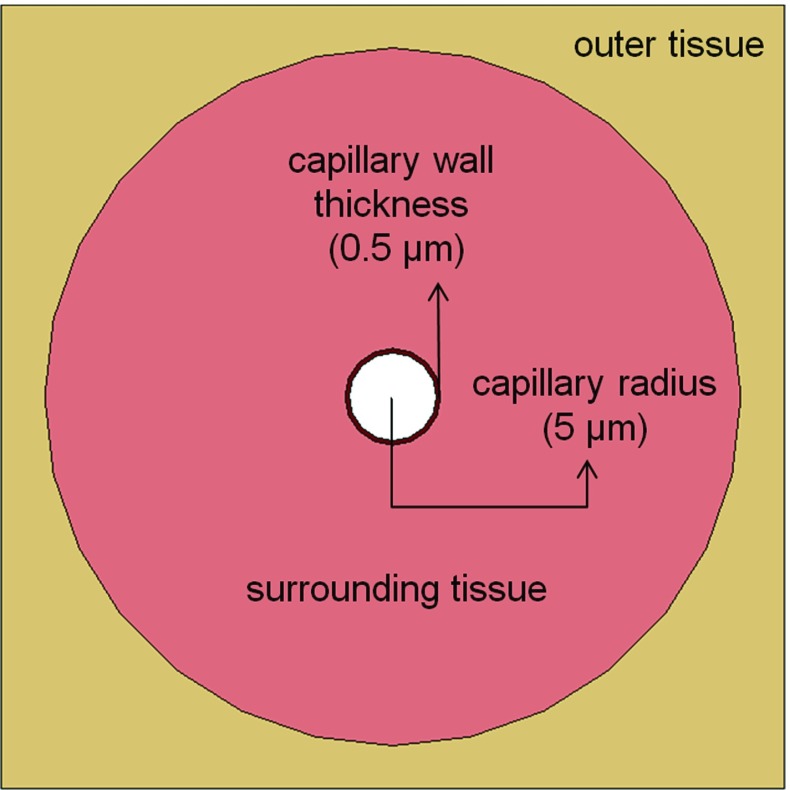
Schematic cross-sectional representation of Krogh cylinder capillary model geometry with capillary wall, surrounding tissue and outer wall

The frequency of the maximum and minimum principal stresses in the ROI was summarised as a histogram distribution (see Fig. 12 in “Appendix”). Values of maximum and minimum principal stresses with the highest probabilities were applied as a complete set of new boundary conditions for the respective meso-scale capillary sub-models. Bed of capillaries in a muscle tissue can be represented using a Krogh cylinder model (Krogh 1919) as a repetitive arrangement of capillaries surrounded by a cylindrical layer of tissue, and the steady-state oxygen supply to surrounding muscle could be studied using a coupled structural and mass-diffusion modelling approach (Popel 1989). In our capillary model, the capillary radius and wall thickness were assumed to be 5 and $0.5\,\upmu \hbox {m}$, respectively, with a $40\,\upmu \hbox {m}$ radius of the Krogh cylinder as surrounding muscle tissue (Popel 1989) (Fig. 5).

#### Structural model of capillary

The surrounding muscle tissue and the capillary wall were modelled as homogeneous hyperelastic materials (Huang et al. 2012) using the Mooney–Rivlin model employed in the meso-scale model; they were considered as homogeneous and continuous hyperelastic materials, which is consistent with the macro-scale model. A constant pressure of 0.5 kPa was applied to the inside of the capillary cavity mimicking a static blood pressure (Ceelen et al. 2008). The specific material parameters used for this model are given in Table 1.

**Fig. 6:**
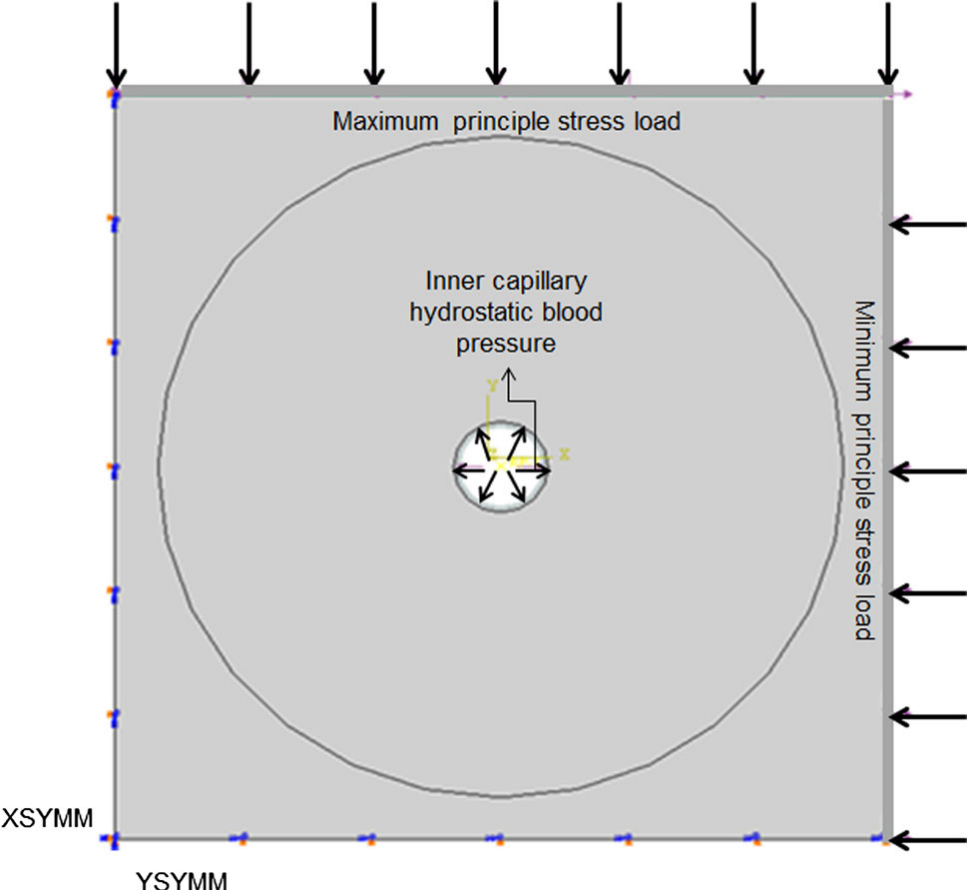
Schematic of boundary conditions for capillary structural model

Most frequent values of the maximum and minimum principle stresses obtained from the macro-scale model were applied as loading conditions to the capillary structural models to represent the effect of deformation of the capillary structure at extreme loading conditions within the ROI (Fig. 6).

#### Mass-diffusion model of capillary

Permeability of capillaries, which allows small molecular-size nutrients and waste products to move between the surrounding tissue and the blood, was modelled using the diffusion model. Relatively, low mass diffusivity of oxygen in the tissue and the mass transfer barrier of the capillary wall result in oxygen concentration gradients in the tissue surrounding the capillary (Fournier 2012) . When oxygen-rich blood contained in the capillary is delivered to the tissue, the difference of oxygen concentration drives diffusion transfer of oxygen to the tissue from the blood through the capillary wall. The following assumptions were made in our diffusion model:

- A tissue surrounds each capillary with the solutes transferred only from that capillary.
- The capillary has a constant radius along its length.
- Within the capillary, the solute is transported primarily by convection in the axial direction and by diffusion in the radial direction. Therefore, diffusion of oxygen within the capillary is not considered in this model; it means that axial diffusion was neglected and only radial diffusion was considered.
- An oxygen consumption rate in the tissue was not considered in the finite-element model.

Numerical solute balance was adopted from the Krogh cylinder model of tissue oxygenation (Magrab 2005) and evaluated using MATLAB (2015b, The MathWorks, Natick, MA, USA) programme to calculate the initial oxygen concentration in blood along the capillary’s axial direction. The capillary considered to have a fixed inlet oxygen concentration $C_{\mathrm{O}_{2,}\mathrm{in}}$ ($120\,\upmu \hbox {M}$) (Magrab 2005) and the external tissue boundary of the capillary model was assumed to have a zero flux. Blood flow within the capillary at a given time point was assumed to be in a steady state, and the oxygen was driven into the tissue components with a constant $C_{\mathrm{O}_{2,}\mathrm{in}}$ and a constant centreline velocity $V_{\mathrm{max}}=0.0005\,\hbox {m}/\hbox {s}$ (Magrab 2005). The decrease in concentration *C* along the length of the capillary was considered in the current study: assuming the cross-sectional layer of our two-dimensional (2D) capillary model as positioned at the middle of a 1-mm-long capillary. The corresponding oxygen concentration was extracted as $40.32\,\upmu \hbox {M}$ at the capillary wall. This value was then used as the initial oxygen concentration for the mass-diffusion model to simulate a steady-state oxygen distribution in the surrounding tissue. The initial concentration of oxygen in surrounding tissue was set to zero in our study.

**Fig. 7:**
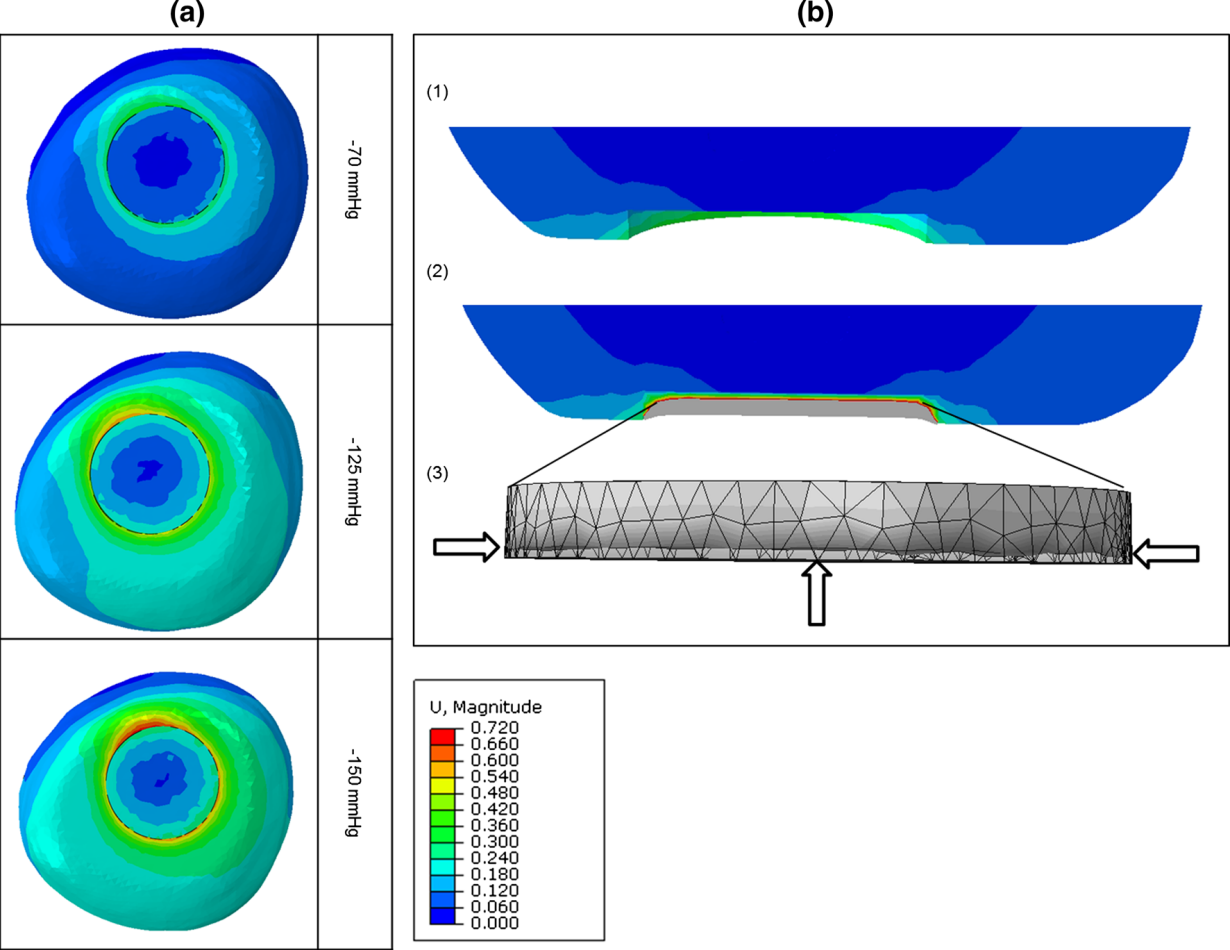
**a** Maximum deformations (in mm) in the transversal plane of the muscle in the wound bed when different negative pressures $-70, -125$, and $-150\,\hbox {mmHg}$ were applied, images from *top* to *bottom*. **b** Frontal view of wound bed deformations at $-125\,\hbox {mmHg}$ without filler (*1*) and with filler (*2*); (*3*) 3D view of filler deformation at $-125\,\hbox {mmHg}$ (*meshed geometry* represents the original shape and the *dark grey area* is the deformed shape)

#### Coupling of structural and mass-diffusion sub-models

The coupling between the structural and mass-diffusion models was achieved by analysing the stress distribution within the structural sub-model first, and then, the equivalent pressure stress field (*p*) obtained from the structural model (see Eq. 2 in Table 1) was incorporated into the same capillary geometry to analyse the mass diffusion within mass-diffusion sub-model. Oxygen diffusion was assumed to be driven by the gradient of the equivalent pressure stress, and the extended form of the Fick’s law (see Eq. 3 in Table 1) was chosen by specifying a nonzero value of the pressure stress factor ($K_\mathrm{P}$). In order to incorporate the pressure field, stress analysis was performed within the capillary structural model, and the pressure field was obtained by specifying the loads and mechanical properties. The structural model input file included an output request to write the incorporated pressure field. Once the mechanical stress analysis was completed, the same geometry was established for a capillary mass-diffusion model, and the obtained pressure field was incorporated into it.

### Post-processing

A change of the oxygenated tissue area surrounding a single capillary relative to the uncoupled reference area was studied. The change in oxygen concentration was calculated for the total area of oxygenation between a previous and new mass-concentration profiles. This new distribution of oxygen concentration in the surrounding tissue was visualised in ABAQUS after the coupling. Then, concentration area profiles with respect to a critical hypoxic fraction were calculated as a range of dimensionless parameters in MATLAB.

## Results

### Effects of negative pressures on tissue macro-deformations

**Fig. 8:**
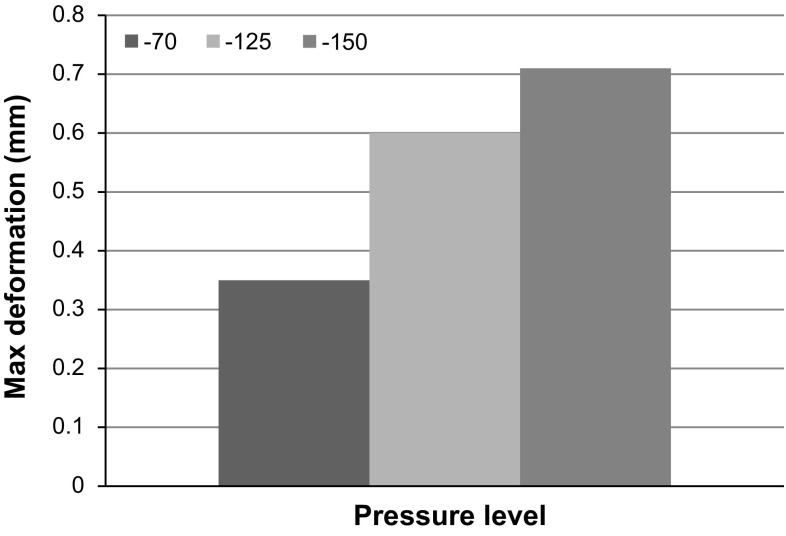
Maximum macro-deformations at various negative pressure levels: $-70, -125$, and $-150\,\hbox {mmHg}$

Mechanical deformations in the tissue exposed to the NPWT were analysed for different pressure levels. Figure 7a illustrates deformation of the muscle part in the transversal plane at various levels of negative pressure. Maximum macro-deformations were observed at the wound edges, and the lateral wound’s width was decreased, in particular in the superficial region, regardless of the pressure applied. In addition, Fig. 8 shows that the increase in pressure levels from $-70$ to $-125$ and $-150\,\hbox {mmHg}$ caused an increase in maximum deformations from 0.3 to 0.6 and $0.7\,\hbox {mm}$, respectively. Figure 7b(1) and (2) shows the frontal plane view of wound bed deformations at $-125\,\hbox {mmHg}$ without and with filler, and a 3D view of filler deformation (Fig. 7b(3)). Apparently, the filler deformed mostly in the outer plane in the direction perpendicular to the transactional plane of the limb with smaller contraction in the radial direction of the cylindrical filler at the lateral surface. Collectively, the computational model of tissue surrounding an excision wound cavity with the presence of filler in a residual limb of trans-femoral amputation exposed to the NPWT demonstrated that the wound filler was compressed against the inner-surface of the wound bed, followed by concentric deformation of the surrounding tissue in the superficial wound edges (see also the video in the supplementary material).

**Fig. 9:**
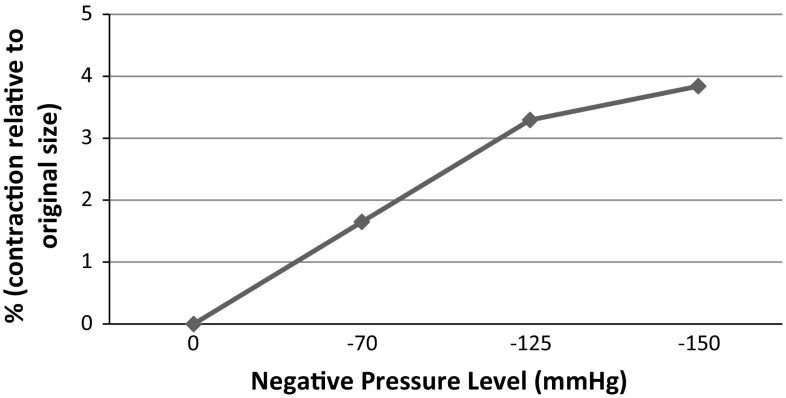
Changes of surface area of cross section of circular wound at various pressure levels

**Fig. 10:**
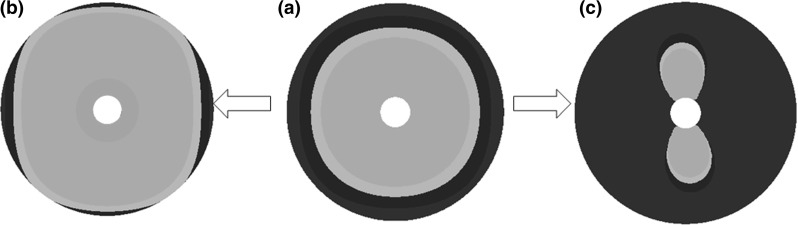
Binary images of coupled mass-diffusion capillary models for MATLAB; a *grey area* represents the area with a critical baseline for sufficient tissue oxygen concentration while the *black area* represents the area below that threshold: **a** uniform oxygen gradient in the tissue surrounding of a single capillary before coupling and **b** and **c** non-uniform gradients in surrounding tissue after coupling

There is also a significant decrease in the wound size upon application of negative pressure due to concentric deformation of the wound edges. In order to quantify the immediate effects of the NPWT on wound contraction, the changes of surface area of cross section of the circular wound were presented as percentage relative to the initial wound surface area before the application of negative pressure (see Fig. 9). The surface area was reduced instantaneously to 3.2% of the original size at $-125\,\hbox {mmHg}$ and 1.6% at $-70\,\hbox {mmHg}$ and 4% at $-150\,\hbox {mmHg}$, respectively. There is a significant change in the wound surface area at $-70\hbox {mmHg}$ and the greatest relative contraction was observed at $-150\,\hbox {mmHg}$, but there was no difference in the reduction in wound area and the extent of reduction reached a plateau at $-125$ and $-150\,\hbox {mmHg}$.

**Fig. 11:**
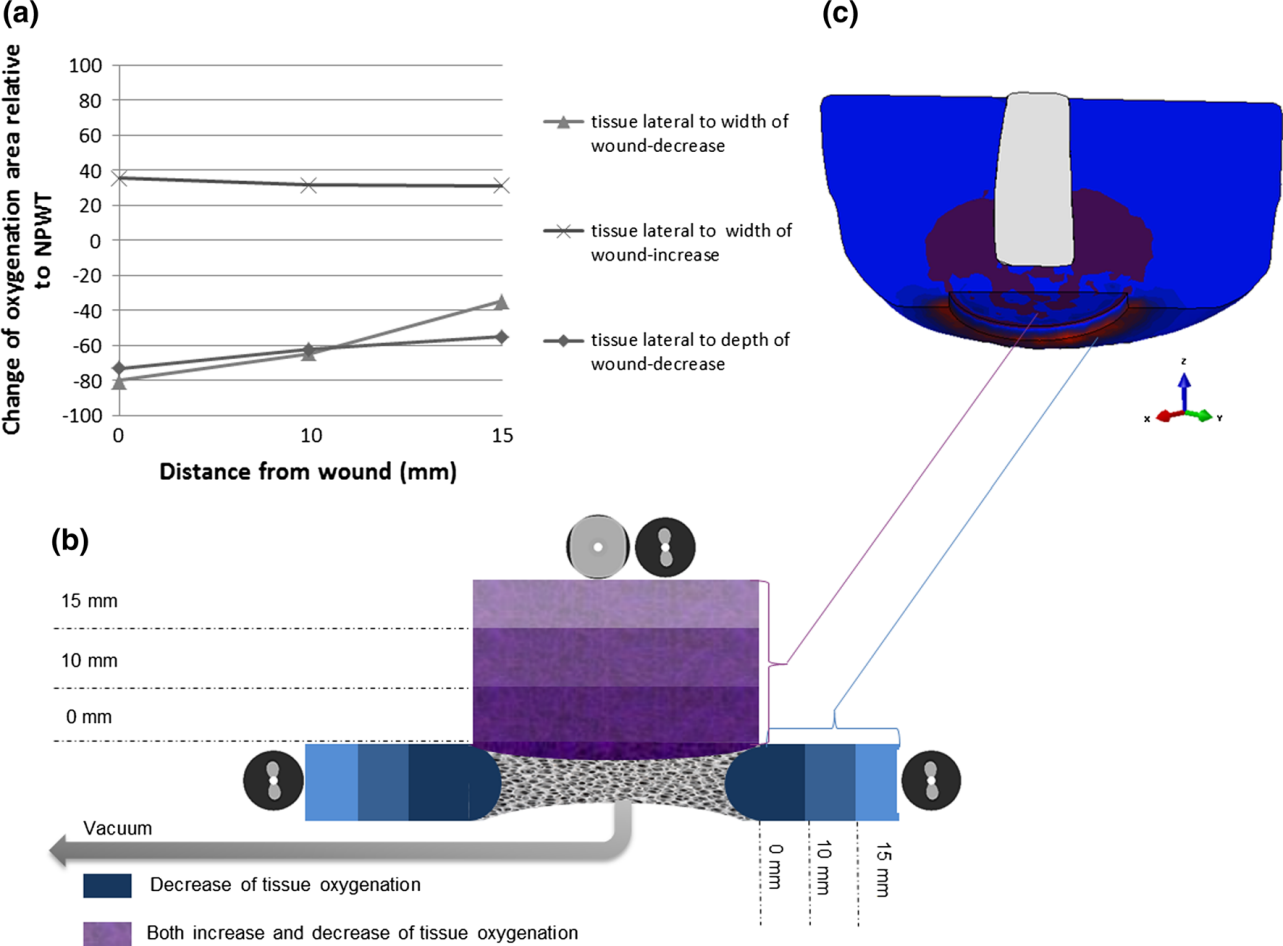
Instantaneous effects of the NPWT on tissue oxygenation area changes at the wound bed cavity, relative to the original values before the NPWT application: **a** lateral to the depth and the width of the wound; **b** schematic description of predicted tissue oxygenation area changes around the wound bed cavity; *purple areas* represent, tissue lateral to the width of the wound and *blue part* represents tissue lateral to the depth of the wound cavity; **c** illustration of the corresponding tissue oxygenation changes in macro-model close to the wound bed area, for 3D demonstration purposes only

### Meso-scale model of oxygen diffusion from capillary

In the meso-scale model, some capillary models experienced compressional and tensional stresses along their two boundaries at the same time while others only experienced compression caused by the corresponding loading conditions defined by the principle stresses in the individual element within the ROI. As a consequence, the capillaries deformed non-uniformly, leading to non-uniform distributions of oxygen gradient in the tissue surrounding the capillary. Relative increases (Fig. 10b) or decreases (Fig. 10c) of tissue oxygenation around the single capillary calculated after coupling the sub-models with respect to the uniformly distributed oxygen gradient (Fig. 10a) are presented as the areas with oxygen concentration either above (grey coloured) or below (black area) the established threshold of $10\,\upmu \hbox {M}$ for sufficient oxygen concentration in the tissue (Magrab 2005).

### Macro-scale tissue oxygenation changes around wound bed

The corresponding changes of the tissue oxygenation area of the ROI directly beneath the filler and adjacent to the wound bed are illustrated for the NPWT at $-125\,\hbox {mmHg}$. Depending on the location of the ROI in respect to the wound bed, two types of changes of oxygenation area were observed, heterogeneous and homogeneous (Fig. 11b, c). Tissue lateral to the depth of the wound cavity (blue areas in Fig. 11b) revealed homogenous patterns of decrease in the oxygenation area. The extent of such decrease was dependent on the distance of the tissue from the wound surface, starting from 73% at the interface of the filler and the wound ($0\,\hbox {mm}$) and then reaching ${\sim }62\%$ and 55% at $10\,\hbox {mm}$ and $15\,\hbox {mm}$, respectively (Fig. 11a). However, tissue lateral to the width of the wound (purple areas in Fig. 11b) exhibits heterogeneous patterns of change, as evidenced by both gradual increase and decrease (Fig. 11b). Those tissue at the interface between the filler ($0\,\hbox {mm}$) experienced larger decreases of up to 80% and then 65 and 35% for deeper tissue at 5 and $10\,\hbox {mm}$ from the wound surface (Fig. 11a). In the meantime, analysis of the same region (purple part in Fig. 11b) also revealed a trend of increases of oxygenation area up to 35% at $0\,\hbox {mm}$ and 31% in deeper tissue at 5 and $10\,\hbox {mm}$ from the surface (Fig. 11a).

## Discussion

The mechanism of the NPWT in wound healing is multifactorial and current results of the NPWT in non-combat-related wounds encouraged military surgeons (Maurya and Bhandari 2016). This technique becomes a preferred method in combat trauma management. For traumatic amputee patients, wound breakdown and survival of the wounded area after wound closure is critical in order to allow rapid rehabilitation and initial prosthetic usage. The concept of these tissue oxygenation studies provides an insight in order to prevent and/or predict possible hypoxic zones of the wound bed, and wound breakdown, depending on the complex shape of combat wounds. Although tissue perfusion studies for non-combat wounds have been explored experimentally and computationally for years, combat wounds have not been studied with computational models yet.

### Tissue macro-deformation and wound contraction

One of the aims of the present study was to examine the macro- deformation upon application of the NPWT, and it provides detailed evidence for the various extents of deformation observed in this simplified wound of trans-femoral amputation. Combat wounds are frequently more complex with significantly greater radius than their thickness (Jeffery 2009). In our models, pulling forces caused by the negative pressure move the superficial tissue wound edges together to a greater extent than inner depths of the edge (Fig. 7). Mechanical effects of the NPWT are important for the entire wound healing process as early changes in the size of the wound have been shown to correlate with final wound healing (Lavery et al. 2007). It is believed that this deformation alters the cytoskeleton resulting in a signalling cascade leading to granulation tissue formation (Malmsjo et al. 2009). This macro-deformation presumably creates shearing forces in the tissue and at the wound-filler interface, promoting tissue formation and facilitating healing (Saxena and Hwang 2004; Torbrand et al. 2010). Shrinkage occurs in three dimensions, and the amount of shrinkage of the wound is determined by the deformability of the surrounding tissues (Orgill et al. 2009). The NPWT in scalp wounds causes minimal contraction of the wound edges with the filler shrinkage occurring mostly perpendicular to the wound surface, which is consistent with our observations based on the developed models (Fig. 7b(3)).

Qualitative observations were published for treatment of patients with ulcers or wounds from open abdomen surgery using the NPWT of different pressure levels and various types of dressing. However, it is not possible to carry out quantitative analysis in clinical practise; hence, outcomes of our study are compared with the published experimental study using rat and porcine subjects. Torbrand et al. (2010) pointed out that a decrease in a lateral wound width during the NPWT was greater in the superficial tissue Studies by Isago et al. (2003) showed $0.3\,\hbox {mm}$ mean reduction at the radius along the wound surface axis of rat models at $-125\,\hbox {mmHg}$, which corresponded to approximately 3% decrease in wound area. Our computational model predicted a trend consistent with their studies (Fig. 9). Additionally, the wound size reduction with the increase in pressure predicted in our macro-model also correlate with observations by Malmsjo et al. (2009). Negative pressure of $-50\,\hbox {mmHg}$ (Malmsjo et al. 2009) or $-75\,\hbox {mmHg}$ (Torbrand et al. 2010; Borgquist et al. 2011b) demonstrated the greatest change in the wound area, with higher levels of negative pressure resulting only in a slight incremental decrease in the wound size. It is not possible to compare directly the levels of maximum macro-deformation and wound size reduction observed in the current study as it depends on the measuring techniques and varies with different subjects and wound shapes.

### Oxygen gradients in the wound tissue

Apart from facilitating wound contraction attributed by macro-deformation of the tissue at the edge of the wound, another mechanism of action of the NPWT is via the introduction of relative hypoxia within the tissue of the wound bed. This would stimulate angiogenesis and promote formation of robust granulation tissue, reducing not only the size but also the depth of the wound. The NPWT has been applied as an effective tool in the treatment of complex extremity wounds caused by major combat trauma. However, there is no research on physicochemical mechanisms of the effects of topical negative pressure on tissue oxygenation, in particular, incorporating the complexity of mechanical deformation of tissue in the residual limb. The tissue oxygenation variation as a consequence of the mechanical stress due to negative pressure would depend on the tissue density and its mechanical properties. The denser and stiffer tissue, i.e. human or porcine skin, would have more resistance to compression and lower maximum deformation than a softer tissue, i.e. those in rodents and rabbits. Such variation would further affect the propagation of stress through layers of tissue and, hence, also the blood flow during the NPWT (Borgquist et al. 2011a).

Principle stress values obtained within the macro-scale models were employed as loading conditions for our capillary simulations, considering the importance of both normal and shear loading on blood perfusion and transcutaneous oxygen levels in human skin as pointed out by Manorama et al. (2010). Consideration of these complex loading conditions in our model allows us to gain insight on patterns of tissue oxygenation perturbed in a 3D model of the residual limb, in response to the local mechanical stress experienced by the wounded muscles. Using porcine wound models, Wackensfors et al. (2004, 2005) reported that the change of microvascular blood flow was dependent on the pressure applied, the distance from the wound edge, and the tissue type. Our meso-scale model also revealed that tissue lateral to the depth of the wound cavity had homogenous patterns of decreased oxygenation, and the extent of such decrease was dependent on the distance of the tissue from the wound surface. This is in agreement with the hypoperfused zone, especially in subcutaneous tissue observed by Wackenfors et al. (2004, 2005).

Apart from this homogenous patterns of oxygenation decrease, it is very intriguing that tissue lateral to the width of the wound revealed heterogeneous patterns of tissue oxygenation change, with domains of both gradual increase and decrease, although still tissue depth dependent. It could imply spatial non-uniform effects of the maximum principal stress due to the resulting intra-tissue pressure, and, hence, the changes of tissue oxygen permeability, within the ROI of the wounded muscles close to the femur at the amputation end. Such heterogeneous patterns were observed in an experimental study of blood flow in tissue close to the wound bed during the NPWT (Malmsjo et al. 2009; Malmsjö et al. 2009b) and also resemble those observed in compartment syndrome in terms of muscle regeneration and pressure stress (Wilkin et al. 2014; Pavan et al. 2017). Furthermore, findings from the current study correlates with diverse published outcomes from *in vivo* experimental work, which encompass from the increased blood flow measured by laser Doppler (Timmers et al. 2005) to the decrease in partial pressure of oxygen in tissue measured with a transcutaneous sensor (Kairinos et al. 2009; Shon et al. 2014)together with tissue depth effects (Erba et al. 2011).

### Parametric studies

As the size of the wound varies within a large span, particularly for the combat wounds, during preliminary studies, it was also investigated whether different wound configurations would result in different maximum deformations in our macro-scale model. A 2D axisymmetric version of the macro-scale model of the lower limb with the similar dimensions was generated, and the effects of geometrical parameters of the wound and the shape of wound edges were studied. The maximum deformation results at $-125\,\hbox {mmHg}$, obtained within this model, were compared with those of the 3D macro-scale model and both revealed similar results. Levels of wound edge maximum deformation were not significantly affected by the shape of the wound edge; only an approximate 4% difference was found between the wounds with round and sharp edges. However, changes in the radius-to-depths ratio of the wound cavity from 1:1 to 2:1 had a significant effect on maximum deformations up to 50%.

### Limitations of the model

In this study, several model assumptions were made due to the prerequisite for simplification, and these should be considered when interpreting our results.

- Many of the wounds caused by the combat-related traumatic injuries have complex shapes with an extensive loss of soft tissues and bony materials (Maurya and Bhandari 2016). In our simplified version of the scenario, the wound shape was assumed as a cylinder-shaped cavity in order to reduce the complexity of the model. Adhesive drape, which seals the wound area over the filler, was not included in the macro-scale model.
- Our simplification of material properties was shown to be accurate enough for the macroscopic model (Portnoy et al. 2009). All evaluations of material properties were based on earlier studies. It may be acceptable to assume a linear property during static loading conditions in our studies; however, for dynamic loading conditions more realistic material properties for soft tissue can be incorporated into the finite-element modelling using nonlinear elements and anisotropic properties.
- Only immediate effects of the NPWT were discussed, and its effects over a prolonged period of time are not the focus of the current study. Hence, changes of mechanical properties of tissues during healing were not considered, as well as evolution of the thickness of the wound bed.
- The porous structure of the filler was not taken into account; therefore, respective microdeformations were not discussed. Influence of the fluid removal process and interstitial pressure was not included.

Admitting these limitations, we have demonstrated that the multiscale models developed can still be applied as a powerful tool to analyse the physiological response of the tissue when the NPWT is applied to more complex wounds. In particular, it would cascade the underling physiochemical mechanisms to scientific-informed wound management practice. Future work would be focused on adapting the established methodology to patient-specific cases of heterotopic ossification, following a blast injury and amputation. A change of tissue oxygenation was proposed as one of relevant iatrogenic factors contributing to the pathophysiology of this disease (Potter et al. 2010).

## Conclusion

We have demonstrated that the developed proof-of-concept multiscale computational models can be applied as tools to gain insights into biomechanical interactions and variations of oxygen gradients of wounded tissue subject to the NPWT. A significant influence of negative pressure levels on both macro-deformations and a change of tissue oxygenation gradients were revealed. The patterns of changes of tissue oxygenation depended on the depth of the tissue, the geometry of the wound, and also the location of tissue plane investigated relative to the wound. With further verification using clinical data as inputs, the developed computational models would aid the stratification of personalised wound management using NPWT for more complex wounds.

### Electronic supplementary material

Below is the link to the electronic supplementary material.

Supplementary material 1 (mov 6240 KB)

## Figures and Tables

**Fig. 12:**
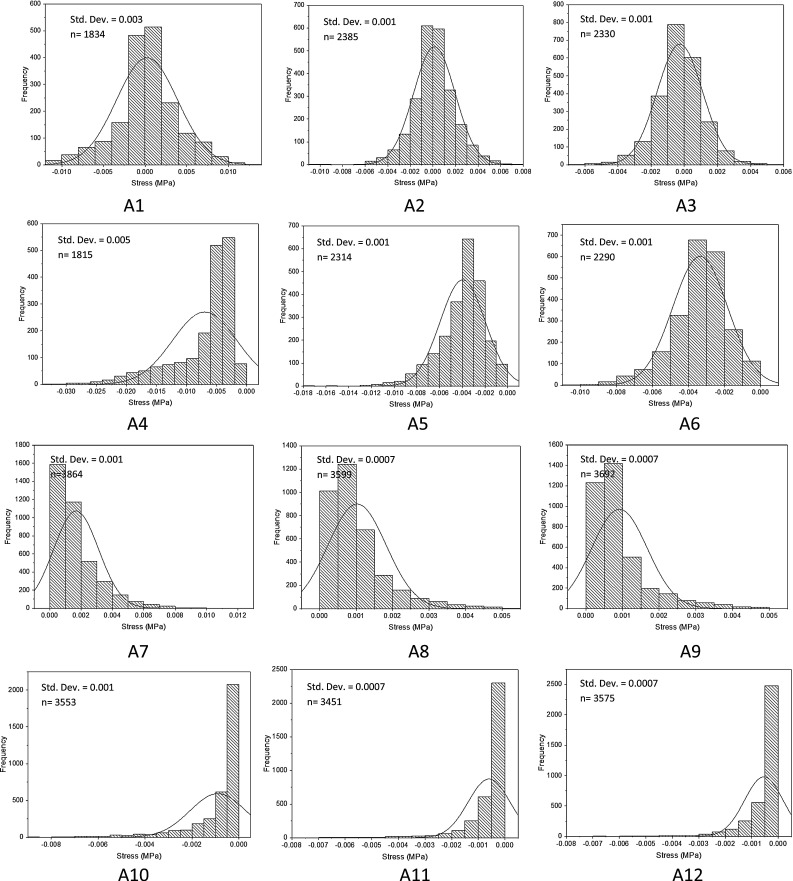
Different histograms of maximum and minimum principal stress distributions caused by NPWT in each cross-sectional ROI area of the macro-scale model: **A1**–**3** Maximum principal stresses cross sections lateral to the width of the wound (0, 10 and $15\,\hbox {mm}$, respectively); **A4**–**6** Minimum principal stresses cross sections lateral to the width of the wound (0, 10 and $15\,\hbox {mm}$, respectively); **A7**–**9** Maximum principal stresses lateral to the depth of the wound (0, 10 and $15\,\hbox {mm}$, respectively); **A10**–**12** Minimum principal stresses cross sections lateral to the depth of the wound (0, 10 and $15\,\hbox {mm}$, respectively)

## Appendix

See Fig. 12.
